# Comment on “Sodium Bicarbonate Therapy in Patients with Metabolic Acidosis”

**DOI:** 10.1155/2015/897517

**Published:** 2015-06-02

**Authors:** Viktor Rosival

**Affiliations:** SYNLAB Department of Laboratory Medicine, Dérer's Hospital, Limbová 5, 833 05 Bratislava, Slovakia

In the paper of Adeva-Andany et al. [1] there are some discrepancies with the literature.

1. The authors write “Evidence that significant harmful effects are derived from metabolic acidosis by itself has not been provided in human beings and therefore the successful management of metabolic acidosis require the therapy of the underlying causative disorder.” According to Edge et al. [2] and Nyenwe et al. [3] metabolic acidosis (= low blood-pH = high concentration of hydrogen ions H_+_) is the immediate cause of decreased level of consciousness including coma: the glycolytic enzyme phosphofructokinase is pH-dependent, as its activity is decreasing with decreasing pH. The consequence is impaired utilisation of glucose in brain cells. Where are published papers denying the results of Edge et al. and Nyenwe et al.?
2. The authors write “Both retrospective and prospective studies have consistently documented that sodium bicarbonate therapy does not improve metabolic responses, biochemical parameters, acid-base balance normalisation, or clinical outcomes among patients with DKA, either children or adults.” In diabetic ketoacidosis, life-threatening is only its most severe stage, coma. If the facts presented in paragraph 1 are correct, thus increase of the very low blood-pH in the comatose patient after infusion of alkalising solution (such as sodium bicarbonate) should result in recovery to normal state of consciousness. And this has been reported in reality: lethality of coma in diabetic ketoacidosis is zero with treatment which includes infusions of alkalising solutions, for example, Umpierrez et al. [4]. Without alkalising solutions, lethality of coma in diabetic ketoacidosis is up to 100%, for example, Basu et al. [5]. Where are published reports on zero lethality of coma in diabetic ketoacidosis without infusions of alkalising solutions?
